# Quantum Physics-Informed Neural Networks

**DOI:** 10.3390/e26080649

**Published:** 2024-07-30

**Authors:** Corey Trahan, Mark Loveland, Samuel Dent

**Affiliations:** U.S. Army Engineer Research and Development Center, Information and Technology Laboratory, 3909 Halls Ferry Rd., Vicksburg, MS 39180, USA

**Keywords:** quantum computing, quantum variational algorithm, quantum machine learning, physics informed neural networks, quantum data-derived methods, quantum algorithms

## Abstract

In this study, the PennyLane quantum device simulator was used to investigate quantum and hybrid, quantum/classical physics-informed neural networks (PINNs) for solutions to both transient and steady-state, 1D and 2D partial differential equations. The comparative expressibility of the purely quantum, hybrid and classical neural networks is discussed, and hybrid configurations are explored. The results show that (1) for some applications, quantum PINNs can obtain comparable accuracy with less neural network parameters than classical PINNs, and (2) adding quantum nodes in classical PINNs can increase model accuracy with less total network parameters for noiseless models.

## 1. Introduction

Quantum computing is rising as an emergent technology with the potential to mitigate hardware bottlenecks and solve problems previously unsolvable on classical computers [1]. We are approaching an era where theory is now transitioning into practice as quantum hardware becomes more available to the scientific community. Quantum software development kits such as Ocean [2], Qiskit [3,4], ProjectQ [5], Strawberry Fields [6], PennyLane [7], and Cirq [8] have facilitated algorithmic design with easy-to-use Python interfaces and quantum computer simulators. While these kits accelerate the design of quantum algorithm prototypes, quantum hardware edges closer to practical use. Although the number of qubits on today’s quantum computers are growing fast, they are still error-prone. However, fault-tolerant quantum computing may be on the horizon as neutral atom arrays have emerged as a promising platform for quantum information processing with logical qubits [9,10].

An area where quantum computing shows promise is in data-driven machine learning applications [11,12,13,14]. Several recent efforts have targeted supervised neural networks (NNs) based on shallow parametrized quantum circuits, as they are prime candidates for near-term applications on noisy quantum computers [15,16,17,18,19]. In supervised learning, a dataset is comprised of inputs and outputs, and the supervised learning algorithm learns how to best map examples of inputs to examples of outputs. In essence, these networks seek to approximate a function represented by data through error minimization between the predicted outputs and the expected outputs during a training process, acting as a universal approximator. This is opposed to discrete classification, another traditional NN implementation. Quantum systems are known to produce atypical patterns that classical systems may not produce efficiently, so it is reasonable to postulate that quantum computers may outperform classical computers on machine learning tasks [14]. Because of this, there has been a surge in quantum machine learning (QML) applications of supervised neural networks. Data-driven QML applications have been widespread, covering the areas of biomedical research [20], computational fluid dynamics [21] and financial modeling [22], for example. Investigations continue into the speed, precision and complexity of both purely quantum and hybrid quantum/classical data-driven networks with respect to potential advantages over classical neural networks (NNs).

While NN function approximation/regression has shown to be a valuable tool for data-rich scenarios, data-only models are not constrained by physics and can perform poorly in sparse- or no-data regions. To build reliable physical models in these regions, physics-informed neural networks (PINNs) can be used [23,24,25]. The implementation of PINNs have led to a series of promising results across a range of problems in computational science and engineering, including fluid mechanics [26,27,28], heat conduction [29], Earth system science [30], power systems [31] and cyber security [32]. PINNs supplement the data-driven loss with partial differential equation (PDE) residuals representing physical conservation principles. For example, consider a time-dependent PDE of the form

(1) $$\begin{array}{c} u_{t}+N\left[u\right]=0,\;\;t\in \left[0,T\right],\;\;x\in \Omega \end{array}$$

subject to initial and boundary conditions

(2) $$\begin{array}{c} u(0,x)=g(x),\;\;x\in \Omega \end{array}$$

(3) $$\begin{array}{c} B\left[u\right]=u_{o},\;\;t\in \left[0,T\right],\;\;x\in \partial \Omega \end{array}$$

where N[·] is a differential operator, and B[·] is a boundary operator corresponding to the equation’s boundary conditions (Dirichlet, Neumann, etc.). If the neural network solution is given by $u_{\theta }\left(t,x\right)$, where θ denotes the tunable parameters of the network (e.g., weights and biases), then the parameterized solution of (1) in residual form is given by

(4) $$\begin{array}{c} R_{\theta }\left(t,x\right)=\frac{\partial u_{\theta }}{\partial t}+N\left[u_{\theta }\right], \end{array}$$

and the PINN is trained on the composite loss function

(5) $$\begin{array}{c} L_{\theta }=L_{ic}\left(\theta \right)+L_{bc}\left(\theta \right)+L_{r}\left(\theta \right), \end{array}$$

where

(6) $$\begin{array}{c} L_{ic}\left(\theta \right)=\frac{1}{N_{ic}}\sum_{i=1}^{N_{ic}}|u_{\theta }\left(0,x_{ic}^{i}\right)-g\left(x_{ic}^{i}\right){|}^{2}, \end{array}$$

(7) $$\begin{array}{c} L_{bc}\left(\theta \right)=\frac{1}{N_{bc}}\sum_{i=1}^{N_{bc}}|B\left[u_{\theta }\right]\left(t_{bc}^{i}.x_{bc}^{2}\right)-u_{o}^{i}{|}^{2} \end{array}$$

(8) $$\begin{array}{c} L_{r}\left(\theta \right)=\frac{1}{N_{r}}\sum_{i=1}^{N_{r}}|R_{\theta }\left(t_{r}^{i},x_{r}^{i}\right){|}^{2} \end{array}$$

**Figure 1:**
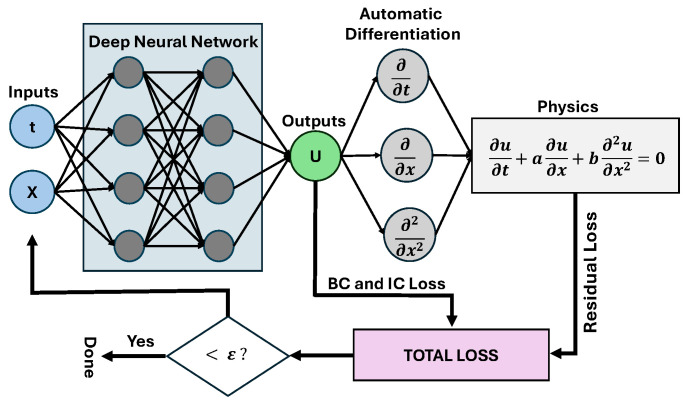
An example classical PINN setup for the solution of an advection–diffusion equation. In this example, *a* and *b* are equation parameters, ε is a user-defined loss tolerance, *x* and *t* are the independent variables (network features), and the neural network solution is given by *u*.

In these equations, $\left\{x_{ic}\right\}$ are $N_{ic}$ initial conditions, $\left\{t_{bc},x_{bc}\right\}$ are $N_{bc}$ boundary conditions and $\left\{t_{r},x_{r}\right\}$ are $N_{r}$ user-defined collocation points over which to evaluate the residual during training. The only data supplied to the loss defined in (5) are the initial and boundary conditions, information normally required for a unique solution to a PDE. External data from field measurements, etc., can also be included in this loss. Figure 1 displays a classical PINN setup for a 2D solution, $u_{\theta }$, of an advection–diffusion equation. For further reading on PINN fundamentals, see [33].

Recently, hybrid physics-informed neural networks (HPINNs) that include both quantum and classical layers have been shown capable of increasing model accuracy when compared to purely classical neural networks for computational fluid dynamics problems [21]. Also recently, HPINNs for the 1D Poisson equation were shown to give good results on continuous variable quantum computers [34]. In this study, we further these efforts by investigating purely quantum physics-informed networks (QPINNS) and HPINNS for solving 1D and 2D PDEs using qubit-based quantum computers and compare their results to classical PINNs. Unlike the applications in [21,34], only the PDE boundary and initial conditions are used for the losses in this study, and non-hybrid QPINNs are investigated. For each PDE, we compare the expressibility and accuracy of the quantum, hybrid and classical networks.

## 2. Quantum and Hybrid PINN Methodology

**Figure 2:**
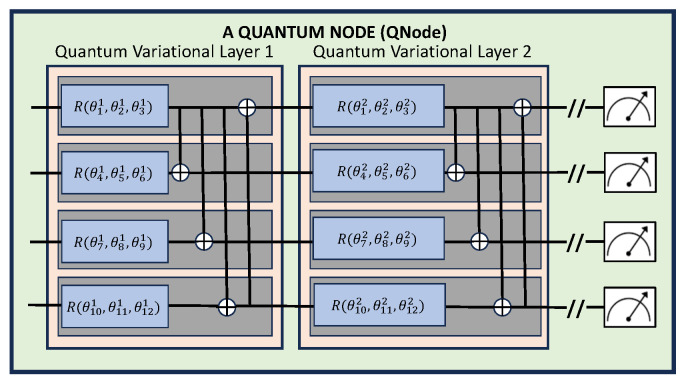
A 4-qubit, strongly entangled, multi-variational layer quantum neural network node. The strongly entangled layers in this network allow for 3D qubit rotational variation, whereas PennyLane’s basic entangled layers replace the three parameter rotations with a single parameter/axis rotation as defined by the user. Here, $\theta_{j}^{\;i}$ represents the *j*th parameter on layer *i*, and *R* is called using three parameter arguments for the qubit’s *x*, *y* and *z* rotations, respectively.

**Figure 3:**
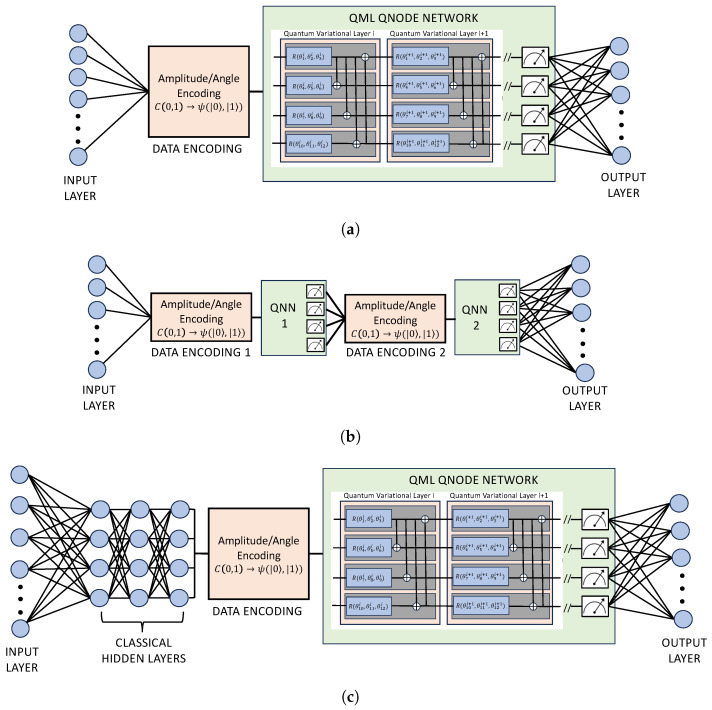
Machine learning model examples for quantum (**a**,**b**) and a hybrid (**c**) neural networks. For each example, the input layers have neurons equal to the feature dimensionality. (**a**) A one-node, multi-variational layer quantum network with strongly entangled qubits. (**b**) A two-quantum-node network with strongly entangled qubits. (**c**) A one-quantum-node, multi-variational layer hybrid network with strongly entangled qubits.

Quantum machine learning (QML) methods are built on quantum neural network nodes, each containing one or more variational layers (see Figure 2) which treat qubit rotations as optimization parameters. For a typical QML setup, the user must specify the number of qubits, quantum nodes and variational layers for each node along with a type of feature encoding. Application results may be notably sensitive to these choices, as detailed in Section 4. However, as can be seen from the three example QML setups given in Figure 3, there are fundamental architectural components to any QML network.

### 2.1. Quantum Variational Layers

At the core of a quantum neural network are some number of quantum variational layers (or blocks) which contain parameterized circuits. These circuits are comprised of gates representing a combination of qubit rotations and entanglers. Note that unlike classical neural networks with contain linear basis functions with nonlinear activations functions, the basis of quantum networks are these trigonometric rotational functions. Increasing the number of qubits on a variational layer not only increases the number of parameters in a linear way, but also can enhance the quantum expressibility of the layer through increasing entanglement via CNOT gates, for example. A common type of entanglement, often called “strong entanglement”, is shown in Figure 2 and consists of single qubit rotations and entanglers. This circuit was inspired by the circuit-centric classifier design given in [15]. An alternative formulation is to replace the full 3D rotations with one-parameter rotations on each qubit. Strongly entangled circuits were found optimal for all but one case in this study. The hybrid Burger’s experiment gave optimal results using single-parameter qubit rotations.

### 2.2. Quantum Neural Network Nodes

As shown in Figure 2, a quantum node contains one or more variational layers and requires qubit measurements before feeding forward into the remaining neural network. Quantum nodes can be placed anywhere in a hybrid network. Our studies have found that number of nodes and their placement can influence the model’s results, particularly as the dimensionality and complexity of the solution increases.

Figure 3a displays a QPINN with one quantum node comprised of four qubits and some number of strongly-entangled variational layers (two are shown). Figure 3b, on the other hand, gives a similar network with two nodes in serial. Although most of the PINN applications in this study only contain one node, there were some QPINN cases where multiple serial nodes converged better (see Section 4.2.2). It is noted that adding nodes can significantly increase the wall-clock time, as encoding and measurements must occur for each quantum node. A quantum node is created in Pennylane’s QML package [7] by adding a *QNode* within a TensorFlow wrapper (see Appendix A).

### 2.3. Input Space and Feature Encoding

The number of features for physics-informed machine learning of partial differential equations is equal to the Cartesian dimensionality, *D*, of the application. In PINN applications, the physics residual is calculated at $x^{D}$ collocation points on the equation domain and added to the total loss. Evaluation of the *D*-feature QPINN/HPINN network at the collocation points is performed in parallel. For quantum networks, encoding a *D*-dimesional collocation point as *D* features can be performed by embedding the data in the basis, amplitude or angle of a qubit. Embeddings impose minimum requirements on the number of qubits in the QPINN along with potential constraints on the size and sign of the feature values. For example, amplitude embedding encodes $2^{n}$ features into the amplitude vector of *n* qubits and requires normalized inputs. Angle embedding, on the the other hand, encodes *n* features into the amplitude vector of *n* qubits.

Both amplitude and angle embedding were investigated for the QPINN and HPINN applications herein, and very little difference was found between the two options. Angle embedding was thus used for the results presented, as this type of embedding did not force feature normalization and required less qubits. It is noted that the 1D and 2D experiments investigated only required a minimum of 1 or 2 qubits for feature encoding and that qubit counts were increased herein solely to study the scalability of network expressivity.

### 2.4. HPINN Design

In the case of hybrid quantum/classic PINNs, users have the same model parameters as above with the added classical network parameters. Figure 3c gives an example of a hybrid neural network. In this figure, the network begins with an input layer and proceeds to the classical hidden layers first. The quantum node follows the hidden layers and produces an output. Our experiments were not sensitive to the position of the quantum nodes in the network; however, our hybrid investigation was limited to only the 2D physics-informed Burger’s application. Placement of the hidden layers may make a notable difference for other applications. Another notable hybrid structure, not shown in this figure or investigated in this effort, is the implementation of parallel quantum/classical networks [35]. For these hybid schemes, both networks process data simultaneously but contribute to the total network loss.

## 3. Quantum Simulator Device Details

TensorFlow v2.16 [36], developed by Google Brain team, Mountain View, California, USA was used to create all the networks used in this study. Noiseless results were obtained using Pennylane’s *default.qubit* device, a state simulator of qubit-based quantum circuit architectures. To include noise in the QPINN experiments, a density matrix formalism was used by implementing Pennylane’s *default.mixed* device. This device supports several noisy channels that are used to describe experimental imperfections. A depolarizing channel was used on this device for all qubits. This channel is modeled by Kraus matrices [37] and requires a user-supplied depolarization probability, p∈[0,1], which is equally divided in the application of all Pauli operations. Note that if p=0, the channel is noiseless. A value of p=0.01 was used for all experiments that included this noise type.

Implementing Pennylane’s *default.mixed* device significantly slowed down the network convergence, prohibitively so for the qubit counts investigated in the HPINN Burger’s equation application. For this case, simulated noise was implemented classically by randomly perturbing the rotational parameters in the quantum variational circuits such that

(9) $$\begin{array}{c} \theta_{i}^{*}=\theta +2\;\beta \;\left(\epsilon -\frac{1}{2}\right) \end{array}$$

where ϵ is a random sample generated from a uniform distribution over [0,1). A value of β=0.03 was used for Burger’s noise simulation.

## 4. QPINN Applications

In order to test the expressibility and accuracy of QPINNs, a 1D spring-mass and 2D Poisson equation was solved using only quantum network components. Solutions for these equations were manufactured that are easily solved with classical PINNs without having to implement sophisticated networks or complex hyper-parameter optimization. Additionally, solutions were chosen that could be modeled with a variational parameter count feasibly calculated with quantum simulators on classical workstations. For the PINN benchmarks, all hyperparameters, such as learning rate, etc., were fully optimized through a series of tests. To test the accuracy of HPINNs, the 2D space-time Burger’s equation was solved with a more complex solution structure. The benchmark used for this study was the optimal benchmark presented in [23,24,25].

### 4.1. Application 1: Spring-Mass System

In this section, QPINNs were used to solve the 1D spring-mass equation given by

(10) $$\begin{array}{c} \frac{d^{2}u}{dt^{2}}+5\;\frac{du}{dt}+6\;u=10\;sin\left(t\right),\;\;\;\;0<t<3 \end{array}$$

(11) $$\begin{array}{c} u\left(0\right)=0,\;\;\;\;\dot{u}\left(0\right)=5 \end{array}$$

The solution to this equation is

(12) $$\begin{array}{c} u(x)=-6\;exp(-3t)+7\;exp(-2t)+sin(t)-cos(t) \end{array}$$

For this application, the QPINN setup given in Figure 3 was used. Adam’s optimizer with a learning rate of 0.02 was found optimal. Only the solution and first-order time-derivative at t=0 were used for the data-driven contribution to the total QPINN loss. The physics-informed contribution was calculated by evaluating the residual of (10) at nc=11 equally distributed collection grid points over the solution domain. Strongly entangled layers were used in the quantum variational circuit, and the max optimization iterations was set to 600 epochs. Lastly, in order to investigate parameter sensitivities, nr=10 runs were used with randomized initial parameters, and the median predicted values were used for the root mean squared error calculations, given by

(13) $$\begin{array}{c} RMSE=\sqrt{\frac{\sum_{i=1}^{nc}{\left({\bar{u}}_{\theta }^{\;i,j}-u\left(x_{i}\right)\right)}^{2}}{nc}}. \end{array}$$

In this equation, ${\bar{u}}_{\theta }^{\;i,j}$ is the median model output over the 10 runs at the $x_{i}$ collocation point for the jth run.

**Figure 4:**
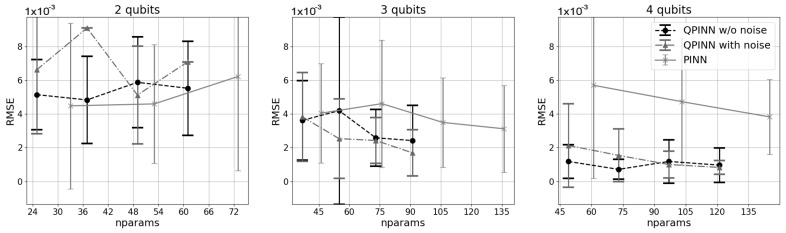
Median and standard deviation RMSE results for the 1D spring-mass problem averaged over 10 runs for a varying number of qubits and variational layers (parameters). QPINN results are shown as black dashed lines with circles (noiseless) and dash-dotted lines with triangles (noise). The solid, gray lines with stars are the classical PINN results. All RMSEs are calculated over the physics-informed collocation points.

To attempt to quantify differences between the QPINN and PINN, the spring-mass problem was solved on a classical TensorFlow network with a similar parameter range, and the root mean squared errors (RMSEs) over the collocation points were compared in Figure 4. In this figure, the number of parameters was increased in the QPINN for a given number of qubits by increasing the number of quantum variational layers in the quantum node. For the classical PINN, the number of neurons per Keras layer was held constant for each of the three plots, and the network parameters were increased by adding Keras layers. We note that multiple classical configurations were investigated, which give the PINN parameter counts listed, and the results shown in this figure were the classical model setups with optimal RMSEs.

As can be seen in Figure 4, the RMSEs show good agreement between the physics-informed results and the analytic solution. Adding quantum noise to the simulation seemed to slightly increase the errors for low qubit counts, but  in general, no trends were found between the noise and noise-free experiments. While the accuracies for the experiments notably improved as the number of qubits were increased, they were not seen to depend as much on the number of variational layers in the quantum ansantz. Classical PINNs performed slightly better when compared to the 2-qubit experiments. However, as the number of qubits was increased, QPINN accuracy was significantly better for a given parameter size, showing promise for the entangled expressibility of the quantum network over classical PINNs. Classical PINNs also had larger RMSE variances for all cases considered.

**Figure 5:**
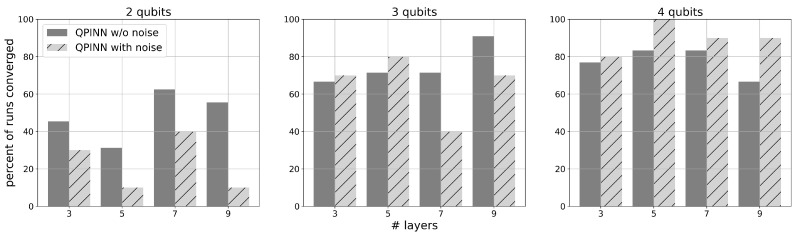
Solution convergence success rates for the 1D spring-mass QPINN problem with increasing qubit and variational layer counts. A successful convergence was achieved when the loss for this problem was less than 0.01 for up to 2000 epochs. Failure occurred more often on low qubit runs.

Some QPINN networks failed to converge for this problem, even as the maximum epochs were increased. Figure 5 displays the convergence rates for each qubit for both noise-free and noisy experiments as the number of layers was increased. Convergence was achieved once the loss reached a user-specified value. These plots show that 2-qubit runs were less successful than higher counts, particularly for noisy calculations. Convergence rates were fairly consistent at about 80% for 3- and 4-qubit runs, and no trends between the noisy and noise-free results were found for these qubit counts. This was the only example where convergence success of the QPINN was this sensitive to the network’s variational parameter initial conditions.

### 4.2. Application 3: 2D Poisson Equation

In this section, QPINN results for two manufactured solutions for the Poisson equation

(14) $$\begin{array}{c} \nabla^{2}u\left(x,y\right)=f\left(x,y\right),\;\;\;\;0<x<1,\;\;\;\;0<y<1. \end{array}$$

are given. For both cases, only the 2D boundary conditions and (14) were supplied to the neural network. The Adam’s optimizer was found to work best with a learning rate of 0.1 for both applications, and the solutions converged well within 500 epochs. A total of 40 equally spaced boundary points were used for the data-driven contribution to the network loss, and the residual was evaluated over a uniform, 11×11 grid of collocation points. Strongly entangled layers were used in the quantum variational circuits with varying qubits and variational layers.

#### 4.2.1. 2D Poisson Quadratic Solution

**Figure 6:**
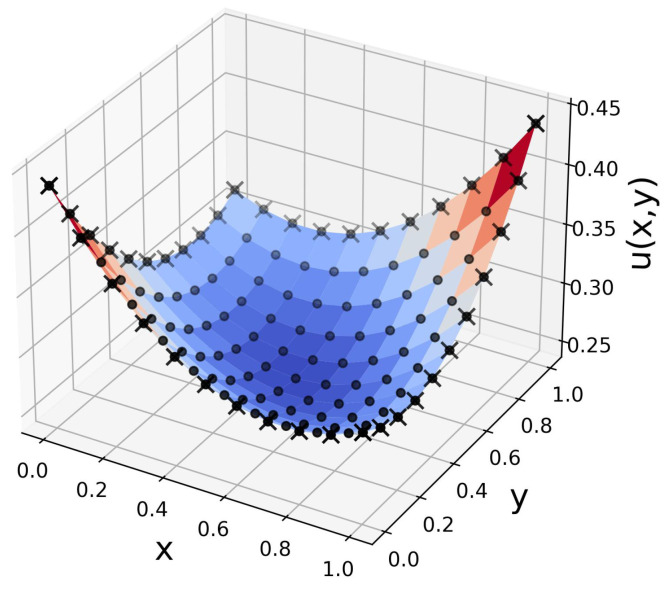
The 2D Poisson quadratic manufactured solution used for QPINN solution. In this figure, the black filled circles are the physics-informed collocation points, and the *x*’s are boundary data for the data-driven loss contributions.

**Figure 7:**
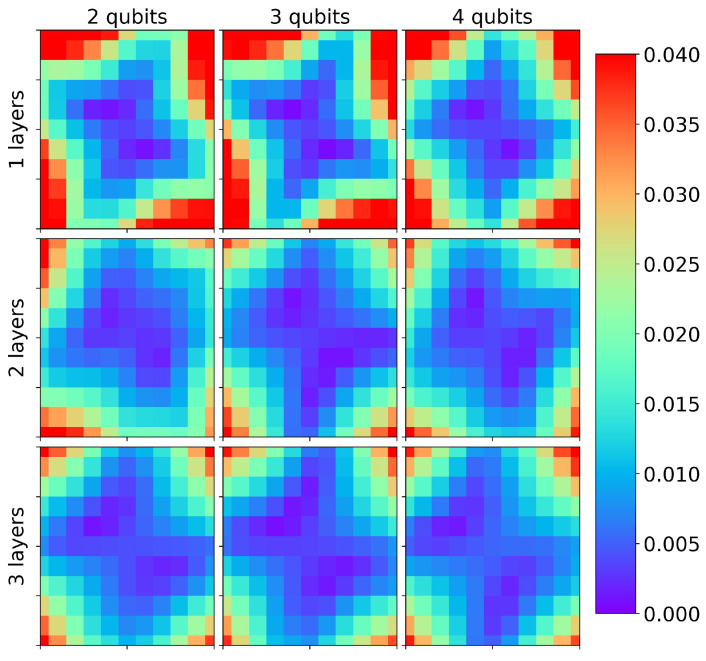
Single-node QPINN 10-run RMSEs at collocation points for the 2D Poisson equation with a quadratic manufactured solution for a range of qubits and nodal variational layers.

In the first test, QPINNs were used to solve for the manufactured Poisson quadratic solution

(15) $$\begin{array}{c} u\left(x,y\right)=\frac{1}{4}\left({\left(x-\frac{1}{2}\right)}^{2}+{\left(y-\frac{1}{2}\right)}^{2}+\left(x-\frac{1}{2}\right)\left(y-\frac{1}{2}\right)+1\right), \end{array}$$

as shown in Figure 6. This solution gives f(x,y)=1 in (14). The QPINN given in Figure 3a was used for a total of 10 runs with varying initial parameters. The 10-run average RMSE errors at the grid collocation points for a range of layers and qubits are shown in Figure 7. The results in the figure include device depolarizing channel noise as described in Section 3. The accuracy of the QPINN solutions were seen to increase with the number of variational layers on the quantum node, as expected, but were not seen to be as sensitive to the number of qubits for this application. This is likely due to the smooth, quadratic solution not requiring high qubit-entangled expressibility.

**Figure 8:**
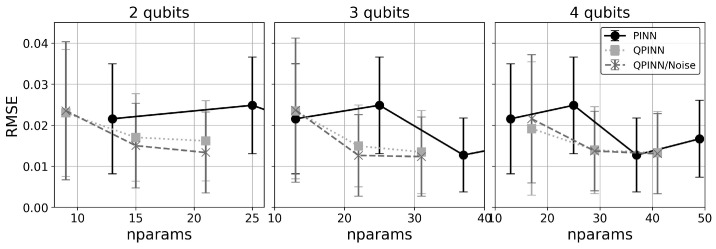
Mean and standard deviations of 10-run collocation point RMSEs for the 2D Poisson quadratic solution versus parameter counts for noiseless QPINNs, QPINNs with noise, and classical PINNs.

**Figure 9:**
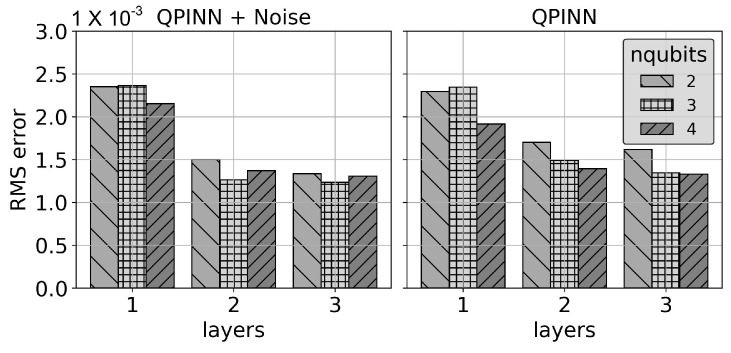
Noise (**left**) and noise-free (**right**) QPINN mean 10-run collocation point RMSE results for the 2D Poisson quadratic solution with increasing variational layer counts.

Figure 8 displays the mean and standard deviation of the collocation point RMSE errors for increasing parameter counts using 2-, 3- and 4-qubit runs along with comparable parameter size classical PINN runs. The classical PINN results for the parameter range shown were obtained by adding 3 neuron layers with “tanh” activation functions between layers. The classical and quantum results gave comparable errors and initial parameter sensitivities for this simple application, though there was a general trend of slightly better QPINN accuracies as the number of qubits was increased. As can be seen from this figure and Figure 9, adding QPINN channel noise slightly increased the accuracy of most experiments. This was not too surprising, as it is well known that Adam’s optimizer handles noise well, and the parameter landscape for this simple problem was likely smooth. This trend is not expected in more complex QPINN applications.

#### 4.2.2. Two-Dimensional Poisson Cubic Solution

**Figure 10:**
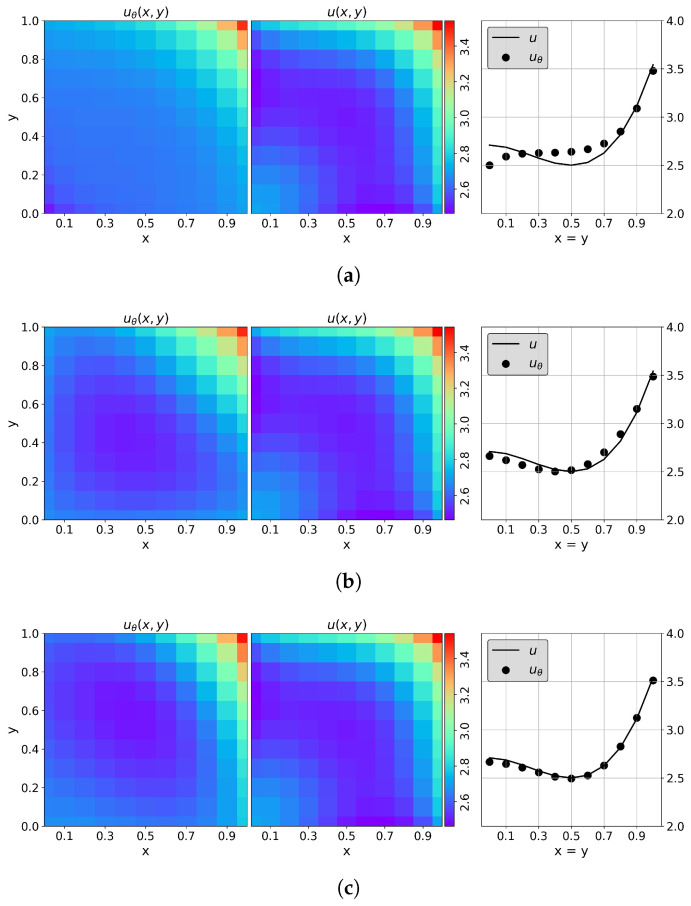
PINN (**a**) and QPINN (**b**,**c**) results for the 2D Poisson equation with a manufactured cubic solution. The QPINN results include device depolarizing channel noise as described in Section 3. In this figure, the left plots are the QML results, the center plots of the analytic solution for comparison, and the right x=y diagonal cross sections of the analytic solution (solid black line) and QML solutions (fill circles). (**a**) Classical PINN results using 2 layers with 5 neurons each for a total of 51 parameters. (**b**) Single quantum node QPINN results comprised of 2 qubits and 4 variational layers for a total of 27 parameters. (**c**) Two quantum node QPINN results comprised of 2 qubits and 2 variational layers for a total of 27 parameters.

**Figure 11:**
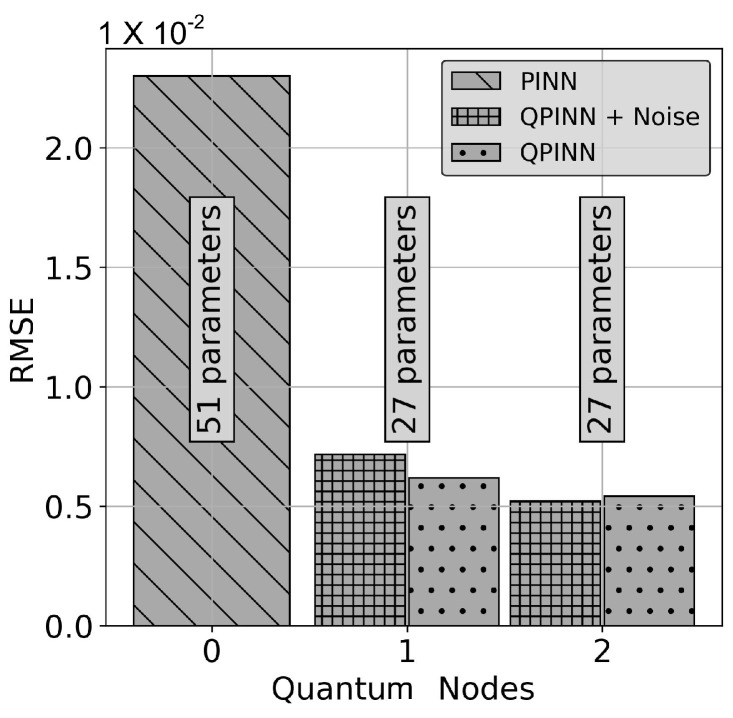
QPINN vs. PINN RMSEs for the Poisson equation with a cubic manufactured solution. These results include device depolarizing channel noise as described in Section 3. The QPINN results shown are for 1- and 2-quantum-node neural networks. The RMSEs calculated in this figure were calculated over 10-run solution averages at the residual collocation points.

In the second Poisson test, the manufactured cubic solution

(16) $$\begin{array}{c} u\left(x,y\right)=\frac{1}{12}\left(2{\left(x-\frac{1}{2}\right)}^{3}+2{\left(y-\frac{1}{2}\right)}^{3}+{\left(x-\frac{1}{2}\right)}^{2}+{\left(y-\frac{1}{2}\right)}^{2}+\left(x-\frac{1}{2}\right)\left(y-\frac{1}{2}\right)+3\right) \end{array}$$

was used so that $f\left(x,y\right)=x+y-\frac{2}{3}$. In this application, the QPINN converged more rapidly and accurately if the solution (and PDE) was scaled by 10. Classical PINNs (shown in Figure 10a) began to converge reasonably well with ≈51 parameters, which were obtained in this figure using 2 layers of 5 neurons each with “tanh” activation functions. A series of 10 runs with varying initial parameters were calculated, and the QPINN grid RMSEs were calculated over the collocation points and averaged over the runs. Multiple quantum node setups were explored in this application. Figure 10b,c display the results for 1 and 2 quantum node networks. From these figures, it can be seen that as quantum nodes are added, the accuracy of the solution slightly improves, even though the total number of network parameters are the same. Figure 11 more clearly shows this improvement. The 2 quantum node QPINN reduced the PINN run-averaged RMSEs by over a 75% with nearly half the parameter counts for this application.

## 5. Hybrid QPINN Application—Burger’s Equation

Burgers’ equation describes the 1D velocity of a moving viscous fluid, and is given by

$$u_{t}+uu_{x}=\nu u_{xx}$$

where u(x,t) is the velocity, and ν is the viscosity of the fluid. For this HPINN application, the spatial domain was set to [−1,1], the temporal domain to [0,1], and $\nu =\frac{0.01}{\pi }$ so that

(17) $$u_{t}+uu_{x}=\frac{0.01}{\pi }u_{xx},$$

along with the homogeneous Dirichlet boundary conditions

(18) $$\left\{\begin{array}{c} u(-1,t)=0\\ u(1,t)=0 \end{array}\right.$$

and initial condition

(19) u(x,0)=−sin(πx),

Note that with this initial condition, a shock is formed at x=0.

The HPINN network used for this application can be seen in Figure 3c. Each of the HPINN models had a structure comprised of TensorFlow Keras layers in a sequential layout, with a model input of two parameters, *x* and *t*, and output of one value, a prediction of u(x,t). The classical PINN model for the Burgers’ equation (taken from [23,24,25]) consisted of an input layer expecting two inputs, nine hidden dense layers with 20 neurons per layer and a hyperbolic tangent activation function, and a final dense output layer with 1 neuron and no activation function. The results from this model were sufficiently accurate to consider it a benchmark model for comparisons. The benchmark model was also used as a basis for adding and tuning the quantum network hyper-parameters. After investigation of different HPINN setups, the final hybrid model utilized a sequential layout starting with four classical dense layers, each with 20 neurons and the hyperbolic tangent activation function. Layer 5 was a classical dense layer with the same number of neurons as the subsequent quantum layer (layer 6), followed by a final dense output layer (layer 7) with one neuron for predicting u(x,t).

All of the quantum layers tested employed one quantum node consisting of an angle embedding, a basic entangling layer, and a measurement. It is noted that for this HPINN experiment, strong entanglement was not optimal and basic entanglement was used in the variational circuits. For experiments that included noise, the classical formulation given in Section 3 was used, as channel noise implementation was prohibitively slow.

Following [23,24,25], each hybrid QPINN was trained using two components: (1) a physics-informed loss based on Burger’s equation over collocation points and (2) a mean squared error (MSE) loss on given data points along the boundaries. For the physics-informed loss, 10,000 random collocation points were generated using the *LatinHypercube* from the *scipy* library on the domain. The loss was calculated using the mean squared residual of (17), given by

(20) $$R=u_{t}+uu_{x}-\frac{0.01}{\pi }u_{xx}$$

All first and second-order derivatives were calculated using TensorFlow’s gradients and GradientTape. For the MSE loss, 50 initial data points at t=0 and 25 data points each on the boundaries located at x=−1 and x=1 were generated with the *LatinHypercube* routine. Data-driven losses were then calculated using the difference between the model’s predictions and the function values at the generated data points on the boundaries using (18) and (19). The Adam’s optimizer was used with a learning rate of 5 × 10^−4^ and a training loop of 2000 epochs.

**Figure 12:**
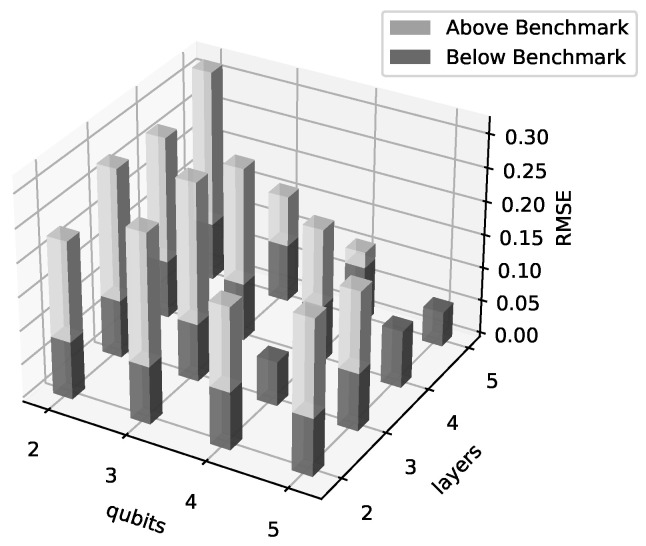
Hybrid QPINN RMSEs for Burger’s equation versus the number of qubits and variational layers in the quantum network. For these results, 5 classical Keras layers preceded the quantum node. The RMSEs were calculated over the residual collocation points solutions averaged over 5 runs. In this figure, the portions of the bars below the classical PINN benchmark are colored a darker gray, while the portions above the benchmark are colored a lighter gray. Bars that fall below the benchmark RMSE are more accurate and are only colored dark gray.

Both the number of qubits and number of variational layers varied from 2 to 5, for a total of 16 hybrid HPINN models tested. The same data and collocation points were used for every model. For comparison, the “exact solution”, provided by [23,24,25], was used to calculate the RMSE of the trained model results. Each model was calculated using five training runs with random parameter intialization, and the run with the median RMSE was recorded as the model for that combination. The noise-free results of these median models are shown in Figure 12. In this figure, the portions of the bars below the classical PINN benchmark are colored a darker gray, while the portions above the benchmark are colored a lighter gray. Bars that fall below the benchmark RMSE are more accurate and are only colored dark gray. The parameter combination with the best RMSE was 5 qubits and 5 layers (5q-5l), with 4q-3l having the second best. Generally, adding more qubits and layers reduced the error, though a notable exception is 4q-3l, which performed exceedingly well compared to the surrounding models throughout the investigation. It should be noted that while many of the hybrid models have a higher error than the benchmark, they were comprised of significantly fewer parameters. For example, the benchmark model has 3441 parameters while the largest, 5q-5l hybrid model gave better results with only 1456 (less than half) parameters. This is a significant improvement over the classical benchmark.

**Figure 13:**
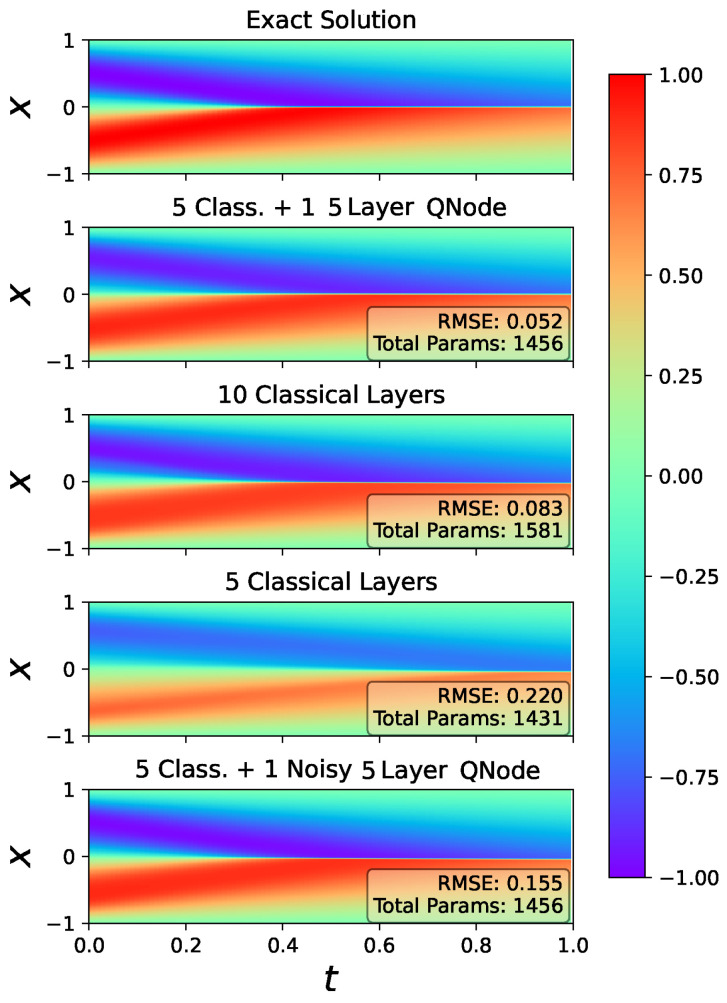
Burger’s equation exact (top plot) and predicted solutions (bottom four plots) for a series of physics-informed models. All RMSEs were calculated over the residual collocation points. In the bottom plot, noise device depolarizing channel noise as described in Section 3 has been added to the HPINN.

For further comparison, the neural network layout of the best hybrid model, 5q-5l, was implemented with only classical layers. The quantum layer was replaced with five classical dense layers, each having five neurons. Note that the models do not perfectly align, as the purely classical quantum-replaced model had 1581 trainable parameters as opposed to the 5q-5l model’s 1456. However, the structure is similar enough for basic comparisons. Additionally, to further show that the quantum layer adds accuracy to the model, a purely classical model with the quantum layer removed was included. These two classical models were given the same training data as the hybrid models and trained over five runs, with the median model solution selected, as before. The predicted solutions from hybrid model 5q-5l and its two derived classical models are shown in Figure 13. The noiseless, 5q-5l HPINN had the lowest RMSE and most closely reflected the exact solution provided in [23,24,25], despite having fewer parameters than the classical quantum-replaced model. Additionally, the accuracy is significantly higher than the quantum-removed model, so there is confidence that the hybrid layer is accurately contributing to the solution and enhancing the accuracy of the problem.

The last plot in Figure 13 shows the HPINN, 5q-5l results with the non-channel, classical noise formulation detailed in Section 3. For this case, the noisy HPINN RMSE was notably worse than the noise-free HPINN calculations though still more accurate than the classical PINNs with a similar parameter count.

## 6. Discussion

In this study, both quantum and quantum/classical, hybrid physics-informed neural networks for PDE solutions were investigated. Four test cases were presented. For the first test case, the 1D spring-mass problem, not only was a purely quantum neural network capable of capturing the PDE solution for a wide range of qubits and variational layers, but there was also an accuracy advantage per parameter over classical networks as the number of qubits was increased. Additionally, less initial parameter sensitivity was found over classical PINNs. For the 2D Poisson problem, manufactured quadratic and cubic solutions were tested. In both cases, there was a greater sensitivity of the models to the initial parameter choice, but classical layers were not required for the physics-informed network to converge. For the quadratic case, only one quantum node was needed in the QPINN and there was a slight accuracy advantage over PINNs for many runs. For the cubic solution, two quantum node networks did show significant accuracy improvement over classical PINNs. Multi-quantum node networks with lower qubit and variational layer counts can help circumvent time-prohibitive and hardware issues when large parameters counts are required. For the cubic problem, greater expressibility was required to capture the solution and higher accuracy was achieved with each quantum node addition. Adding depolarization channel noise to the QPINNs did not seem to significantly affect their solutions.

In the last application, a space-time, hybrid physics-informed neural network was used to solve Burger’s equation for viscous flow. It was found that noiseless hybrid PINNs can notably increase the accuracy of classical PINNs. In the results presented herein, there was a ≈62% increase in accuracy of the noiseless HPINN over a PINN with more than 100 additional parameters. It is also noted that for this case, strongly-entangled variational layers were not optimal. When classically simulated noise was included, the HPINN advantage greatly diminished for this application. Future work will include investigating different implementations of quantum noise and their affect on HPINN solutions.

The results presented herein show that in the context of physics-informed neural networks, qubit-based quantum variational circuits can offer an accuracy advantage in the near term. In some cases, QPINNs can be applied standalone, while in more complex applications, hybrid or quantum co-processor NNs are still required. The focus of this study was to investigate the expressibility of both QPINNs and HPINNs versus PINNs. Wall-clock timings were not emphasized for these experiments, as the quantum network components were significantly slower than their classical counterparts. For example, the noiseless 2D Poisson QPINN results, calculated by a Mac computer with a 12-core, 2.7 GHz Xeon processor, took nearly four times as long as a classical PINN with a similar number of parameters. For some experiments, this was prohibitively the case when device simulated channel noise was included and/or as the number of quantum nodes was increased. While measurement sampling will always slow-down quantum calculations, an era of fault-tolerant quantum computing is likely to come. As these machines become available, the computational overhead for dealing with decoherence related noise will diminish. Additionally, there is no need to model noise on quantum hardware, even today, so this calculation overhead is irrelevant. While fault-tolerant hardware advances work to close the wall-clock gap between quantum and classical neural networks, it is uncertain as to whether the potential parameter space savings offered by the enhanced quantum expressibility will be substantial enough to make up for quantum to classical hardware connections, space transformations and sampling times required by both QPINNs and HPINNs.

## Figures and Tables

**Figure A1:**
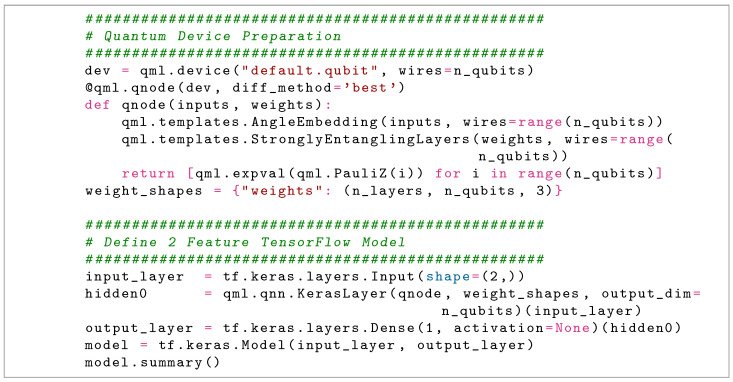
Example TensorFlow wrapped QNN.

## Data Availability

All source code used for the results found in this study can be found online: https://github.com/trahancj/QPINNs.git, accessed on 22 July 2024.

## Appendix A. Example TensorFlow/QNode Setup

A basic TensorFlow/QML wrapper for a purely quantum network with two features can be given as (Figure A1)

In this QML setup, only one quantum layer is used with no classical layers other than the input and output layers, neither of which have parameters or activation functions. For general solution ranges, an input layer tanh-activation may be required to scale the solution. We note that Pennylane’s QML package can be implemented for pure quantum networks without TensorFlow, and similar results were obtained when this was completed. TensorFlow wrapping, on the other hand, allowed for easy extension to hybrid networks and a closer comparison to classical networks.

In Figure A1, strongly-entangled layers over the user-supplied number of qubits are used for the quantum variational circuit. The variational layers within the network are comprised of 3D qubit rotational gates, as shown in Figure 2. This type of network requires three variational parameters per qubit. An alternative is PennyLane’s basic-entangled layer, where only one axis rotation is parameterized.

In Figure A1, the network features are angle-encoded. Because of this, it is necessary that all feature values have a domain of 0≤x≤2π. Note that angle encoding requires 1 qubit per feature dimension.
